# Validity and Reliability of the Hungarian Version of the Pain Self-Efficacy Questionnaire Among Women with Endometriosis and Chronic Pelvic Pain

**DOI:** 10.1089/whr.2024.0109

**Published:** 2025-01-14

**Authors:** Zsófia Kovács-Szabó, Alexandra Makai, Pongrác Ács, Márta Hock

**Affiliations:** ^1^ University of Pécs Institute of Physiotherapy and Sports Science, Pécs, Hungary.; ^2^ Doctoral School of Health Sciences, Faculty of Health Sciences, University of Pécs, Pécs, Hungary.; ^3^ Physical Activity Research Group, Szentágothai Research Center, University of Pécs, Pécs, Hungary.

**Keywords:** endometriosis, pelvic pain, validity, reliability, self-efficacy

## Abstract

**Background:**

Our study aimed to perform Hungarian cross-cultural adaptation and assess the reliability and validity of the Pain Self-Efficacy Questionnaire (PSEQ) in women diagnosed with endometriosis and chronic pelvic pain.

**Methods:**

The current study was conducted in Hungary among women aged 18–50 (34.39 ± 6.68 years). We examined the reliability of the Hungarian version of PSEQ (PSEQ-HU) by applying internal consistency and test–retest evaluations. Confirmatory factor analysis was used to determine the construct validity of the PSEQ-HU, while Spearman’s rank correlation coefficient established the convergent validity using the 36-Item Short-Form Health Survey, numeric rating scale (NRS), Pain Catastrophizing Scale, and Perceived Stress Scale. To determine discriminant validity, two groups were created based on NRS (0–4 no or mild pain, 5–10 moderate or strong pain). The results were analyzed using IBM SPSS version 28.0 software with a significance level of *p* ≤0.05.

**Results:**

A total of 262 women participated in this study. The Cronbach’s α was 0.97, and the intraclass correlation coefficient (ICC) values showed adequate reliability (ICC = 0.94. 95% confidence interval 0.88–0.97) for the PSEQ-HU. Based on the Spearman’s correlation coefficients the convergent validity showed significant results (*r* = 0.22–0.63; *p* ≤0.001).

**Conclusion:**

We concluded that the PSEQ is a reliable and valid measurement among Hungarian women with endometriosis-related pelvic pain.

## Introduction

Endometriosis is a chronic, progressive, inflammatory gynecological disease that affects more than 170 million women worldwide.^1^ In the United States 11% of women and approximately every 10th women worldwide including Hungary suffer from endometriosis.^2,3^

The prevalence of endometriosis in Hungary, as reflected in outpatient data, increases with age with 275 per 100,000 among 20–29-year-olds, 695 per 100,000 among 30–39-year-olds, and 439 per 100,000 among 40–49-year-olds. In Hungary according to a recent study 1.91 million Euro (EUR) was spent on endometriosis treatment in 2019.^4^

The impact of endometriosis on work productivity is significant, as women with this condition experience an average loss of 11 hours of work per week.^5^ Endometriosis is frequently associated with painful syndromes such as cyclical or noncyclical chronic pelvic pain (CPP). CPP is a common chronic pain syndrome that affects a significant number of women, especially those with endometriosis.^6^ The global prevalence of CPP varies from 5% to 43% in females and 2% to 17% in males.^7^

CPP associated with endometriosis significantly decreases health-related quality of life in affected women, often resulting in elevated levels of fatigue, anxiety, and depression.^1,8–10^ Consequently, a comprehensive questionnaire is necessary to assess coping strategies and self-efficacy among women with CPP.

Psychological factors play an essential role in coping with chronic pain, and self-efficacy has been identified as an important resilience factor in managing pain, moreover, it has a significant effect on functioning and coping with chronic pain.^11^ Pain-related self-efficacy has been proven to be an even more important determinant of disability than pain intensity, fear of movement, pain duration, or anxiety.^12,13^ A lower level of pain-related self-efficacy was identified with higher pain intensity,^14^ while a higher level of pain-related self-efficacy was associated with greater physical functioning and physical activity participation. Pain-related self-efficacy has been confirmed to be negatively associated with stress and pain catastrophizing among patients with chronic pain.^15^

There are specific scales for measuring self-efficacy related to different diseases, such as the Arthritis Self-Efficacy Scale^16^ and the Rheumatoid Arthritis Self-Efficacy Scale.^17^ There are also scales for chronic pain self-efficacy, such as the Chronic Pain self-Efficacy Scale^18^ and the Pain Self-Efficacy Questionnaire (PSEQ). The PSEQ^19^ is an extensively researched and reliable tool for evaluating pain-related self-efficacy in subjects with chronic pain according to a 2019 Delphi study.^20^

The original version of the PSEQ was developed and validated in English by Nicholas et al.^19^ and has been tested on samples with chronic pain.

Excellent reliability and validity have been demonstrated in different languages such as Japanese,^15^ Dutch,^21^ Hebrew,^22^ Yoruba,^23^ Italian,^24^ Arabic,^25^ Catalan,^26^ Persian,^27^ Canadian-French,^28^ and Brazilian.^29^ As the PSEQ is still not available in the Hungarian language, the translation and cultural adaptation of the PSEQ is important because it could be used in various aspects of CPP-related outpatient or inpatient care as well as in Hungarian clinical studies.

The present study aimed to translate the PSEQ into Hungarian language through a process of cross-cultural adaptation and to test its measurement properties on a Hungarian sample of patients presenting with both CPP and endometriosis.

## Methods

The current study was a cross-sectional online survey, distributed *via* social media using endometriosis support groups in Hungary between January 2023 and May 2023.

Women aged 18–50 living in Hungary, with both self-reported medical diagnosis of endometriosis (*via* surgery, ultrasound, or MRI-Magnetic Resonance Imaging examination) and self-reported CPP “pain symptoms perceived to originate from pelvic organs/structures typically lasting more than 6 months,”^30^ were voluntarily recruited for the study. Pregnant women, women not diagnosed with endometriosis, and women under 18 and above 50 years of age were excluded from the study. We asked 39 participants to fill in the questionnaire 2 weeks after the initial testing according to the COnsenus-based Standards for the selection of health status Measurement INstruments (COSMIN) guideline and other relevant studies^31–33^ (COSMIN checklist is in Supplementary Data S1).

### Original pain self-efficacy questionnaire

The self-administered PSEQ is a specific questionnaire developed to evaluate pain-related self-efficacy in individuals with chronic pain. It assesses the impact of self-management interventions and confidence in one’s ability to function despite experiencing chronic pain. The questionnaire includes 10 questions rated on a 7-point Likert scale. The score is calculated by summing the responses, the maximum score is 60 showing excellent pain-related self-efficacy. Cutoff points of the PSEQ scores are: low (ranging from 0 to 11), medium (ranging from 12 to 32), and high (ranging from 33 to 60).^34^ The original English version of the questionnaire showed excellent internal consistency (Cronbach’s α **=** 0.92) and test–retest reliability.^19^

### Pain Catastrophizing Scale

The Pain Catastrophizing Scale (PCS)^35^ is a validated, self-assessment questionnaire, consisting of 13 statements containing different thoughts and feelings that may be experienced when enduring pain. The items are divided into the categories of rumination (items 8–11), magnification (items 6, 7, and 13), and helplessness (items 1–5 and 12), with each item scored on a 5-point scale. The overall score has a range from 0 to 52. Higher scores indicate a higher level of pain catastrophizing. The Hungarian version of the PCS^36^ has demonstrated good internal consistency: Cronbach’s α = 0.74–0.93 (rumination subscale = 0.79, magnification subscale = 0.74, helplessness subscale = 0.93).

### 36-Item Short-Form Health Survey

The 36-Item Short-Form Health Survey^37^ (SF-36) is scored in two steps: first, raw scores are summed for each domain, yielding scores between 0 and 100, with 0 representing the lowest and 100 indicating the highest health-related quality of life. In step two, items in the same scale are averaged together to create eight domains: physical functioning (10 items), bodily pain (2 items), role limitations due to physical health problems (4 items), role limitations due to emotional problems (3 items), emotional well-being (5 items), social functioning (2 items), energy/fatigue (4 items), and general health perceptions (6 items).

During the cross-cultural adaptation of the SF-36 the Hungarian version of the SF-36^38^ showed excellent internal consistency: Cronbach’s α = 0.93.

### Perceived Stress Scale-10

The Perceived Stress Scale (PSS)-10^39^ is a self-administered questionnaire developed to assess stress levels and “the degree to which individuals appraise situations in their lives as stressful.” The scale includes 10 questions, consisting of five answer options related to frequency, varying from 0 to 4. Higher scores indicate higher stress levels, with 40 indicating the highest possible score. The Hungarian version of the PSS-10^40^ had good internal consistency (Cronbach’s α = 0.85).

### Numeric pain rating scale

The scale^41^ is a widely used and valid instrument in the literature that aims to assess the subjective level of pain on a scale of 0–10, where 0 is no pain at all and 10 is the maximum possible pain. In the current study, the participants were asked to rate their average and maximum pain levels in the last 4 weeks and their actual pain intensity.

### Validation process

#### Translation and cultural adaptation process

Translation and cross-cultural adaptation were completed through a multistage process.^42^ After receiving consent from the original investigator of the PSEQ for the translation, cross-cultural adaptation, and validation into the Hungarian language, the linguistic validation was carried out according to the relevant guidelines.

1. In this, the PSEQ was forward-translated from English to Hungarian by two independent translators. The first translator was aware of the concept of the questionnaire and as a physiotherapist had a clinical background, while the second translator was not informed about the questionnaire, and as an English teacher, did not have a clinical perspective. Then a synthesis between translations was made to confirm the equivalence with the original version.
2. The back-translation of the PSEQ into the original English language was performed by an independent physiotherapist with high English proficiency.
3. We had a committee composed of a medical doctor, an English teacher, a physiotherapist, and a patient with endometriosis, which created the prefinal version of the questionnaire.
4. The prefinal version was piloted to 15 Hungarian women suffering from endometriosis and CPP and they were asked to report back on the readability, cultural relevance, and comprehensiveness of the translated version. Only minor changes were suggested, so the final Hungarian version of the self-administered PSEQ-HU was accepted.

Following the creation of the final version of the questionnaire, we evaluated the following psychometric properties of the PSEQ-HU: content validity, structural validity, internal consistency, test–retest reliability, convergent validity, and discriminative validity.

### Statistical analysis

#### Reliability

Internal consistency assesses the degree to which the items within an instrument are interconnected, indicating their strength of association (homogeneity). Cronbach’s α was used to verify internal consistency and values between 0.70 and 0.95 are considered adequate.^43^ Test–retest reliability was examined using the intraclass correlation coefficient (ICC). ICC values of 0.75–1.00 were interpreted as excellent.^44^

#### Validity

Structural validity was explored by score distributions and floor and ceiling effects; the latter was considered present if more than 15% of the sample achieved the lowest (floor) or highest (ceiling) possible score.

Our study aimed to confirm the structural validity of the instrument using confirmatory factor analysis (CFA) and reliability statistics such as comparative fit index (CFI), Tucker–Lewis index (TLI), root mean square error of approximation (RMSEA), and 90% confidence interval (CI). A ratio of <3 between the chi-square test and degrees of freedom (dfs), CFI and TLI of 0.90 or more, and RMSEA values of 0.08 or less indicated a good model fit. We calculated factor loadings for each item and identified loadings below 0.5 for potential item reduction.^45^

Convergent validity of the PSEQ was assessed by using Spearman’s rank correlation analysis between the total score of the PSEQ and the NRS, SF-36, and PCS.^24^ PSS correlation was classified as moderate (0.3 < *r* > 0.69) or strong (*r* > 0.7).^46^

For the examination of discriminant validity, based on the average scores of NRS over the past 4 weeks, we created two groups (0–4 no or mild pain and 5–10 moderate and severe). The Mann–Whitney U test was used to test discriminative validity to examine the differences in PSEQ scores between patients with mild or moderate/severe pain.^47^

Descriptive statistics were applied to establish sample characteristics, central tendency estimates, and variability of the dataset. These data are presented as means, standard deviations (SDs), or percentages (%).

Data analysis was performed using IBM SPSS 28.0 software and IBM SPSS AMOS 29.0 (SPSS Inc., Chicago, IL) version. The level of significance was set at *p* < 0.05.

## Results

**FIG. 1.**
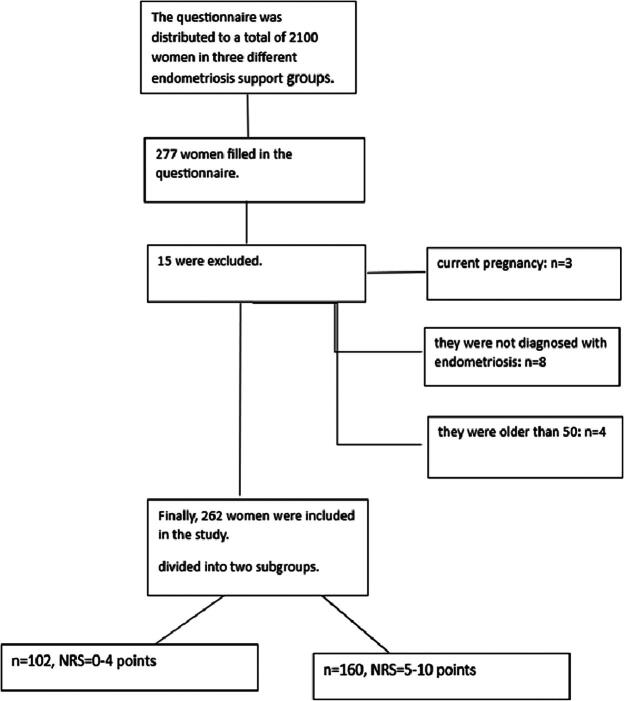
Flowchart of the recruitment process of the sample. NRS, numerical rating scale.

Initially, 277 participants were recruited and 15 were excluded, therefore 262 adult women with both endometriosis and CPP (mean age 34.39 ± 6.68) participated in the study (Fig. 1). A subgroup of 39 participants was asked to fill out the survey twice, for a second time 14 days after the initial testing. The whole sample’s profile and sociodemographic characteristics are presented in Table 1.

**Table 1.** Sample Characteristics

Variable	Final sample *n* = 262 (*n*; %)
1. Marital status	
Living alone	34 (13.1%)
Living with partner	94 (36.2%)
Married	127 (48.8%)
Divorced	5 (1.9%)
2. Educational level	
Elementary school	4 (1.5%)
Secondary school	106 (40.6%)
Higher education	151 (57.9%)
3. Work category	
Sedentary	154 (59.7%)
Light physical	56 (21.7%)
Heavy physical	7 (2.7%)
Standing	27 (10.5%)
Unemployed	14 (5.4%)
4. Residence	
City	112 (42.9%)
Village	43 (16.5%)
Administrational center	41 (15.7%)
Capital	65 (24.9%)
7. Pelvic pain duration (years)	9.23 ± 7.7

The internal consistency of the PSEQ- HU was excellent with a Cronbach’s α of 0.97 (Table 2). The test–retest reliability of the PSEQ-HU in a sample of 39 women yielded excellent results (ICC: 0.939. 95% CI: 0.884–0.968).

**Table 2.** Internal Consistency of the PSEQ-HU

Item	Mean (SD)		Corrected item-total correlation	Cronbach’s α when deleted
PSEQ-HU 1	3.60	1.81	0.823	0.964
PSEQ-HU 2	4.11	1.78	0.864	0.962
PSEQ 3	3.76	1.97	0.854	0.962
PSEQ 4	3.87	1.80	0.861	0.962
PSEQ 5	4.01	1.85	0.869	0.962
PSEQ 6	3.57	1.93	0.890	0.961
PSEQ 7	2.73	2.26	0.700	0.969
PSEQ 8	3.56	2.01	0.871	0.962
PSEQ 9	3.52	2.03	0.892	0.961
PSEQ 10	3.44	1.97	0.876	0.962
PSEQ-HU Cronbach’s α = 0.966				

PSEQ-HU, Hungarian version of the Pain Self-Efficacy Questionnaire; SD, standard deviation.

The floor and ceiling effects were insignificant; 0.7% and 8.9% of the participants scored the lowest and highest possible scores, respectively. Table 3 shows the mean and SD values for the different questionnaires.

**Table 3.** The Descriptive Results of the Instruments

Outcomes	Mean (SD)
Pain self-efficacy (PSEQ- HU)	36.18 ± 17.04
Current pain intensity, NRS	4.09 ± 3.02
Perceived stress level, PSS	30.92 ± 8.72
Pain catastrophizing, PCS	27.46 ± 14.24
Health-related quality of life, SF-36	
Physical functioning	73.38 ± 23.59
Role limitations due to physical health	43.87 ± 41.61
Role limitations due to emotional problems	50.83 ± 44.39
Energy/fatigue	45.06 ± 14.03
Emotional well-being	46.16 ± 14.45
Social functioning	62.26 ± 32.35
Pain	46.33 ± 28.11
General health	44.93 ± 23.74

NRS, numerical rating scale; PCS, Pain Catastrophizing Scale; PSS, Perceived Stress Scale; SF-36, 36-Item Short-Form Health Survey.

**FIG. 2.**
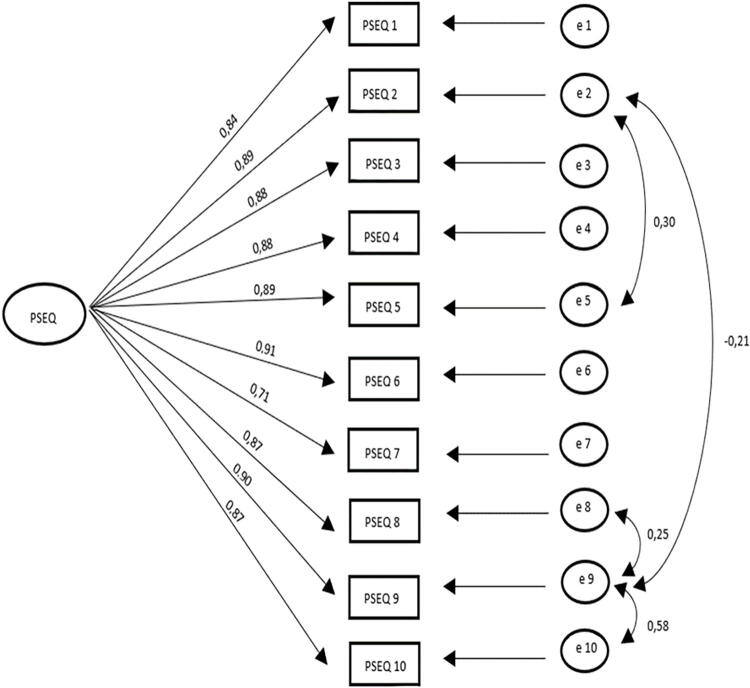
Confirmatory factor analysis of PSEQ-HU: initial model. PSEQ-HU: Hungarian version of the Pain Self-Efficacy Questionnaire.

The CFA supported the one-factor structure of the PSEQ-HU for the sample we used (RMSEA = 0.070; TLI = 0.981; CFI = 0.987; chi-square = 70.597; df = 31; *p* < 0.001; chi-square/df = 2.277). All items converged meaningfully onto the scale as predicted with significant standardized factor loadings (all *p* < 0.01) ranging from 0.71 to 0.91. The applied modification indices between the different items were presented in Figure 2.

Table 4 presents the correlations between the PSEQ-HU and the other measurement tools. We found weak to moderate correlations between SF-36 subscales and the PSEQ-HU total score and significant negative moderate correlations between the PCS, PSS, and PSEQ-HU total scores.

**Table 4.** Convergent Validity of the PSEQ-HU and the Other Measurement Tools (*n* = 262)

Questionnaire	Spearman’s rank correlation coefficients with total score with total score of PSEQ HU (r. p) (*n* = 262)
*SF-36*	
*Physical functioning*	0.539^**^
*Role functioning physical*	0.552^**^
*Social functioning*	0.560^**^
*Bodily pain*	0.626^**^
*Role functioning emotional*	0.427^**^
*Emotional well-being*	0.221^**^
*Energy/Vitality*	0.520^**^
*General health perception*	0.427^**^
*PSS*	−0.404^**^
*PCS*	−0.544^**^

Level of significance.

^**^ *p* < 0.001.

During the examination of discriminant validity, we applied the Mann–Whitney U test, and the test results indicated a significant difference in the mean scores of the PSEQ-HU between the two groups formed based on the current NRS (*p* < 0.001). The “no or mild pain” group (45.35 ± 15.19) and the “moderate or severe pain” group (30.41 ± 15.58) showed significant (*p* < 0.001) differences in the PSEQ-HU mean value scores.

## Discussion

Our study aimed to translate and cross-culturally adapt the PSEQ into the Hungarian language and examine its reliability and validity in a sample of women with endometriosis who reported CPP. The PSEQ itself has been used in other studies examining CPP.^48^ We examined the psychometric properties of the Hungarian version of PSEQ-HU and the cross-cultural adaptation and validation process was similar to those in the relevant literature. The reliability and validity of the PSEQ-HU were in congruence with the results of other similar studies.

Consistent with other studies, CFA supported the one-factor structure of the PSEQ in the current study. By deleting each item, the Cronbach’s α remained stable. No significant floor and ceiling effects were found, similar to other studies like the Italian^24^ version.

The PSEQ-HU showed good psychometric properties with good reliability and validity. The PSEQ-HU showed excellent internal consistency (Cronbach’s α = 0.97), in congruence with the Hebrew^22^ (Cronbach’s α = 0.97), Italian^24^ (Cronbach’s α = 0.94), Persian^27^ (Cronbach’s α = 0.92), Dutch^21^ (Cronbach’s α = 0.92), and the original validation^29^ (Cronbach’s α = 0.92) studies. The PSEQ-HU had excellent test–retest reliability.

The PSEQ-HU demonstrated high test–retest reliability, in congruence with other validation studies of the PSEQ. We used 2 weeks between the initial test and retest as suggested by the relevant literature.^31,32^

Convergent validity was confirmed in this study. The study showed that PSEQ-HU was significantly and negatively associated with measures of pain catastrophizing, higher stress, and pain levels.^49^ Lower level of pain-related self-efficacy was correlated with a higher level of pain catastrophizing, perceived stress, and pain. The correlation between the PSEQ-HU and the PCS was moderate (*r* = −0.54), similar to that in the Japanese (*r* = −0.49) and Italian (*r* = −0.42) validation studies. Correlations with NRS: current study (*r* = −0.48) and Italian (*r* = −0.39).

Our findings that higher health-related quality of life was associated with higher self-efficacy are consistent with the results of other cross-cultural adaptations and validations.

Among the SF-36 domains we found a significant moderate positive correlation between physical functioning (*r* = 0.54) and similar moderate correlations in other studies, Dutch (*r* = 0.32), Amharic (*r* = 0.38), and Persian (*r* = 0.31). Bodily pain had the strongest correlation with PSEQ-HU in the current study (*r* = 0.63) while in other studies it was moderately associated: Amharic (*r* = 0.51) and Japanese (*r* = 0.45). Similar to the Amharic (*r* = 0.40), Persian (*r* = 0.51) and Japanese (*r* = 0.31) validation studies we also found significant moderate correlations between the PSEQ-HU and the general health domain of the SF-36 (*r* = 0.427). Vitality was also significantly correlated with the PSEQ-HU in our study (*r* = 0.52), similar to the Persian (*r* = 0.51) and Japanese (*r* = 0.46) results. Regarding social functioning, we found moderate correlations (*r* = 0.56), similar to the Dutch (*r* = 0.49), Persian (*r* = 0.43), and Japanese (*r* = 0.43) validations. A moderate correlation was found between PSEQ-HU and the role limitations due to the physical health domain of SF-36 (*r* = 0.55), following the Amharic (*r* = 0.32), Persian (*r* = 0.33), and Japanese (*r* = 0.41) validation studies of the PSEQ. Role limitations due to mental health showed a moderate significant correlation in our study (*r* = 0.42), the Japanese (*r* = 0.41) and Persian (*r* = 0.34) studies also showed similar moderate correlations. Only the mental health and emotional well-being domain showed a weak correlation in the current study (*r* = 0.22), while in other studies moderate correlations were found (Amharic [*r* = 0.36], Persian [*r* = 0.42], Japanese [*r* = 0.40]).^15,21^

In the present study, we can observe that higher health-related quality of life values correspond to greater pain-related self-efficacy.

The results of the current study indicated a significant difference in the mean scores of the PSEQ-HU between the two groups^47^ formed based on the NRS values of the participants (*p* < 0.001).

Although the current study has several strengths, it also has some limitations. First, all patients were recruited from three thematic social media groups, and the sample was not representative. The diagnosis of endometriosis and CPP was self-reported. The number of participants was 262, which is an average number. We performed test–retest reliability on 39 participants. This is the first time that this measurement tool has been validated for CPP and endometriosis.

## Conclusions

The current study indicated that the PSEQ-HU is a valid and reliable instrument for evaluating pain-related self-efficacy in individuals with CPP. The translation and cultural adaptation of the PSEQ-HU could contribute to further studies on CPP, pain-related self-efficacy, and factors influencing pain-related self-efficacy.

The PSEQ-HU may be a useful tool for determining the effects of different treatment methods on chronic pain and pain-related self-efficacy. Additionally, the questionnaire can contribute to measuring the effectiveness of a pain management program, in order to provide even more effective, patient-centered care in the Hungarian population diagnosed with endometriosis and CPP, as pain-related self-efficacy is an important predictor of overall health beliefs and it is in connection with the health-related quality of life.

## Data Availability

All data generated or analyzed in this study were included in this published article. The data presented in this study are available upon request from the corresponding authors. Due to privacy concerns the unedited (or raw) data are not publically available.

## Abbreviations Used

ASES: Arthritis Self-Efficacy Scale
CFA: Confirmatory Factor Analysis
CFI: Comparative Fit Index
CI: Confidence Interval
COSMIN: COnsenus-based Standards for the selection of health status Measurement INstruments
CPP: Chronic Pelvic Pain
CPSES: Chronic Pain self-Efficacy Scale
EUR: Euro
ICC: Intraclass Correlation Coefficient
MRI: Magnetic Resonance Imaging
NRS: Numeric Rating Scale
PCS: Pain Catastrophizing Scale
PSS: Perceived Stress Scale
RMSEA: root mean-square error approximation
SD: Standard Deviation
SF-36: 36 Item Short-Form Health Survey
TLI: Tucker-Lewis Index
