# Supplementary material for: Validity and Reliability of the Hungarian Version of the Pain Self-Efficacy Questionnaire Among Women with Endometriosis and Chronic Pelvic Pain
Source: Womens Health Rep (New Rochelle). 2025 Jan 14;6(1):69–77. doi: 10.1089/whr.2024.0109 (PMC11773173; doi:10.1089/whr.2024.0109)
Supplement: Supplementary Data S1 [file whr.2024.0109_supplememtarydatas1.pdf]

Box General requirements for studies that applied Item Response Theory (IRT) models

		yes	no	?
1	Was the IRT model used adequately described? e.g. One Parameter Logistic Model (OPLM), Partial Credit Model (PCM), Graded Response Model (GRM)	☐	☑	
2	Was the computer software package used adequately described? e.g. RUMM2020, WINSTEPS, OPLM, MULTILOG, PARSCALE, BILOG, NLMIXED	☐	☑	
3	Was the method of estimation used adequately described? e.g. conditional maximum likelihood (CML), marginal maximum likelihood (MML)	☐	☑	
4	Were the assumptions for estimating parameters of the IRT model checked? e.g. unidimensionality, local independence, and item fit (e.g. differential item functioning (DIF))	☐	☐	☑

X

	yes	no	?
Was the percentage missing items described?	☑	☐	
Was described how missing items were handled?	☑	☐	
Was the sample size included in the internal consistency analysis adequate?	☑	☐	☐
Were there any important flaws in the design or methods of the study?	☑	☐	

Box A. Internal consistency

		yes	no	?
1	Does the scale consist of effect indicators, i.e. is it based on a reflective model?	☑	☐	☐
	Design requirements	**yes**	**no**	**?**
2	Was the percentage of missing items given?	☑	☐	
3	Was there a description of how missing items were handled?	☑	☐	
4	Was the sample size included in the internal consistency analysis adequate?	☑	☐	☐
5	Was the unidimensionality of the scale checked? i.e. was factor analysis or IRT model applied?	☑	☐	
6	Was the sample size included in the unidimensionality analysis adequate?	☑	☐	☐
7	Was an internal consistency statistic calculated for each (unidimensional) (sub)scale separately?	☑	☐	☐
8	Were there any important flaws in the design or methods of the study?	☐	☑	
	Statistical methods	**yes**	**no**	**NA**
9	for Classical Test Theory (CTT): Was Cronbach's alpha calculated?	☑	☐	☐
10	for dichotomous scores: Was Cronbach's alpha or KR-20 calculated?	☐	☐	☑
11	for IRT: Was a goodness of fit statistic at a global level calculated? e.g. χ^2, reliability coefficient of estimated latent trait value (index of (subject or item) separation)	☑	☐	☐

Box B. Reliability: relative measures (including test-retest reliability, inter-rater reliability and intra-rater reliability)

	Design requirements	yes	no	?
1	Was the percentage of missing items given?	☑	☐	
2	Was there a description of how missing items were handled?	☑	☐	
3	Was the sample size included in the analysis adequate?	☑	☐	☐
4	Were at least two measurements available?	☑	☐	
5	Were the administrations independent?	☑	☐	☐
6	Was the time interval stated?	☑	☐	
7	Were patients stable in the interim period on the construct to be measured?	☑	☐	☐
8	Was the time interval appropriate?	☑	☐	☐
9	Were the test conditions similar for both measurements? e.g. type ofadministration, environment, instructions	☑	☐	☐
10	Were there any important flaws in the design or methods of the study	☐	☑	

	Statistical methods	yes	no	NA	?
11	for continuous scores: Was an intraclass correlation coefficient (ICC) calculated?	☑	☐	☐	
12	for dichotomous/nominal/ordinal scores: Was kappa calculated?	☐	☐	☑	
13	for ordinal scores: Was a weighted kappa calculated?	☐	☐	☐	☑
14	for ordinal scores: Was the weighting scheme described? e.g. linear, quadratic	☐	☐	☑	

Box C. Measurement error: absolute measures

Design requirements		**yes**	**no**	**?**
1	Was the percentage of missing items given?	☑	☐	
2	Was there a description of how missing items were handled?	☑	☐	
3	Was the sample size included in the analysis adequate?	☑	☐	☐
4	Were at least two measurements available?	☑	☐	
5	Were the administrations independent?	☑	☐	☐
6	Was the time interval stated?	☑	☐	
7	Were patients stable in the interim period on the construct to be measured?	☑	☐	☐
8	Was the time interval appropriate?	☑	☐	☐
9	Were the test conditions similar for both measurements? e.g. type of administration, environment, instructions	☑	☐	☐
10	Were there any important flaws in the design or methods of the study?	☐	☑	
Statistical methods		**yes**	**no**	**?**
11	for CTT: Was the Standard Error of Measurement (SEM), Smallest Detectable Change (SDC) or Limits of Agreement (LoA) calculated?	☐	☑	

Box D. Content validity (including face validity)

General requirements		**yes**	**no**	**?**
1	Was there an assessment of whether all items refer to relevant aspects of the construct to be measured?	☐	☐	☑
2	Was there an assessment of whether all items are relevant for the study population? (e.g. age, gender, disease characteristics, country, setting)	☑	☐	☐
3	Was there an assessment of whether all items are relevant for the purpose of the measurement instrument? (discriminative, evaluative, and/or predictive)	☑	☐	☐
4	Was there an assessment of whether all items together comprehensively reflect the construct to be measured?	☑	☐	☐
5	Were there any important flaws in the design or methods of the study?	☐	☑	

Box E. Structural validity

		yes	no	?
1	Does the scale consist of effect indicators, i.e. is it based on a reflective model?	☑	☐	☐

Design requirements		yes	no	?
2	Was the percentage of missing items given?	☑	☐	
3	Was there a description of how missing items were handled?	☑	☐	
4	Was the sample size included in the analysis adequate?	☑	☐	☐
5	Were there any important flaws in the design or methods of the study?	☐	☑	

Statistical methods		yes	no	NA
6	for CTT: Was exploratory or confirmatory factor analysis performed?	[redacted]		
7	for IRT: Were IRT tests for determining the (uni-) dimensionality of the items performed?	☐	☑	☐

Box F. Hypotheses testing

Design requirements		yes	no	?
1	Was the percentage of missing items given?	☑	☐	
2	Was there a description of how missing items were handled?	☑	☐	
3	Was the sample size included in the analysis adequate?	☑	☐	☐
4	Were hypotheses regarding correlations or mean differences formulated a priori (i.e. before data collection)?	☑	☐	☐*

		yes	no	NA
5	Was the expected *direction* of correlations or mean differences included in the hypotheses?	☑	☐	☐
6	Was the expected absolute or relative *magnitude* of correlations or mean differences included in the hypotheses?	☑	☐	☐
7	for convergent validity: Was an adequate description provided of the comparator instrument(s)?	☑	☐	
8	for convergent validity: Were the measurement properties of the comparator	☑	☐	

	instrument(s) adequately described?			
9	Were there any important flaws in the design or methods of the study?	☐	☑	
Statistical methods		**yes**	**no**	**NA**
10	Were design and statistical methods adequate for the hypotheses to be tested?	☑	☐	☐

Box G. Cross-cultural validity

	Design requirements	yes	no	?
1	Was the percentage of missing items given?	☑	☐	
2	Was there a description of how missing items were handled?	☑	☐	
3	Was the sample size included in the analysis adequate?	☑	☐	☐
4	Were both the original language in which the HR-PRO instrument was developed, and the language in which the HR-PRO instrument was translated described?	☑	☐	
5	Was the expertise of the people involved in the translation process adequately described? e.g. expertise in the disease(s) involved, expertise in the construct to be measured, expertise in both languages	☑	☐	
6	Did the translators work independently from each other?	☑	☐	☐
7	Were items translated forward and backward?	☑	☐	☐
8	Was there an adequate description of how differences between the original and translated versions were resolved?	☑	☐	
9	Was the translation reviewed by a committee (e.g. original developers)?	☑	☐	
10	Was the HR-PRO instrument pre-tested (e.g. cognitive interviews) to check interpretation, cultural relevance of the translation, and ease of comprehension?	☑	☐	
11	Was the sample used in the pre-test adequately described?	☑	☐	
12	Were the samples similar for all characteristics except language and/or cultural background?	☑	☐	☐
13	Were there any important flaws in the design or methods of the study?	☐	☑	
	Statistical methods	**yes**	**no**	**NA**
.4	for CTT: Was confirmatory factor analysis performed?	☑	☐	☐
.5	for IRT: Was differential item function (DIF) between language groups assessed?	☐	☐	☑

Box H. Criterion validity

	Design requirements	yes	no	?
1	Was the percentage of missing items given?	☑	☐	
2	Was there a description of how missing items were handled?	☑	☐	
3	Was the sample size included in the analysis adequate?	☑	☐	☐
4	Can the criterion used or employed be considered as a reasonable 'gold standard'?	☑	☐	☐
5	Were there any important flaws in the design or methods of the study?	☐	☑	
	Statistical methods	**yes**	**no**	**NA**
6	for continuous scores: Were correlations, or the area under the receiver operating curve calculated?	☑	☐	☐
7	for dichotomous scores: Were sensitivity and specificity determined?	☐	☐	☑

Box I. Responsiveness				
Design requirements		**yes**	**no**	**?**
1	Was the percentage of missing items given?	☑	☐	
2	Was there a description of how missing items were handled?	☑	☐	
3	Was the sample size included in the analysis adequate?	☑	☐	☐
4	Was a longitudinal design with at least two measurement used?	☑	☐	
5	Was the time interval stated?	☑	☐	
6	If anything occurred in the interim period (e.g. intervention, other relevant events), was it adequately described?	☑	☐	
7	Was a proportion of the patients changed (i.e. improvement or deterioration)?	☑	☐	
Design requirements for hypotheses testing		**yes**	**no**	**?**
For constructs for which a gold standard was not available:				
8	Were hypotheses about changes in scores formulated a priori (i.e. before data collection)?	☑	☐	☐*
		**yes**	**no**	**NA**
9	Was the expected *direction* of correlations or mean differences of the change scores of HR-PRO instruments included in these hypotheses?	☑	☐	☐
10	Were the expected absolute or relative *magnitude* of correlations or mean differences of the change scores of HR-PRO instruments included in these hypotheses?	☐	☐	☑
11	Was an adequate description provided of the comparator instrument(s)?	☑	☐	
12	Were the measurement properties of the comparator instrument(s) adequately described?	☑	☐	
13	Were there any important flaws in the design or methods of the study?	☐	☑	
Statistical methods		**yes**	**no**	**NA**
14	Were design and statistical methods adequate for the hypotheses to be tested?	☑	☐	☐
Design requirement for comparison to a gold standard		**yes**	**no**	**?**
For constructs for which a gold standard was available:				
15	Can the criterion for change be considered as a reasonable gold standard?	☑	☐	☐
16	Were there any important flaws in the design or methods of the study?	☐	☑	

Statistical methods		**yes**	**no**	**NA**
17	for continuous scores: Were correlations between change scores, or the area under the Receiver Operator Curve (ROC) curve calculated?	☑	☐	☐
18	for dichotomous scales: Were sensitivity and specificity (changed versus not changed) determined?	☐	☐	☑

**Box J. Interpretability**		**yes**	**no**	**?**
1	Was the percentage of missing items given?	☑	☐	
2	Was there a description of how missing items were handled?	☑	☐	
3	Was the sample size included in the analysis adequate?	☑	☐	☐
4	Was the distribution of the (total) scores in the study sample described?	☑	☐	
5	Was the percentage of the respondents who had the lowest possible (total) score described?	☑	☐	
6	Was the percentage of the respondents who had the highest possible (total) score described?	☑	☐	
7	Were scores and change scores (i.e. means and SD) presented for relevant (sub) groups? e.g. for normative groups, subgroups of patients, or the general population	☑	☐	
8	Was the minimal important change (MIC) or the minimal important difference (MID) determined?	☐	☑	
9	Were there any important flaws in the design or methods of the study?	☐	☑	

Box Generalisability box

			yes	no	NA
	Was the sample in which the HR-PRO instrument was evaluated adequately described? In terms of:				
	1	median or mean age (with standard deviation or range)?	☑	☐	
	2	distribution of sex?	☑	☐	
	3	important disease characteristics (e.g. severity, status, duration) and description of treatment?	☑	☐	☐
	4	setting(s) in which the study was conducted? e.g. general population, primary care or hospital/rehabilitation care	☑	☐	
	5	countries in which the study was conducted?	☑	☐	
	6	language in which the HR-PRO instrument was evaluated?	☑	☐	
7	Was the method used to select patients adequately described? e.g. convenience, consecutive, or random		☑	☐	
			**yes**	**no**	**?**
8	Was the percentage of missing responses (response rate) acceptable?		☑	☐	☐
